# Supplementary material for: Effect of TiO2 Nanoparticle on Bioaccumulation of ndl-PCBs in Mediterranean Mussels (Mitilus galloprovincialis)
Source: Animals (Basel). 2023 Mar 30;13(7):1208. doi: 10.3390/ani13071208 (PMC10093413; doi:10.3390/ani13071208)
Supplement: Supplementary file 1 [file animals-13-01208-s001.zip › animals-2243960-supplementary.pdf]

Table S1. Instrumental mass spectrometry settings for target compounds.

PCB congeners	Quantitative SRM			Confirmation SRM		
	Precursor ion (m/z)	Product ion 1 (m/z)	Collision energy (eV)	Precursor ion (m/z)	Product ion 1 (m/z)	Collision energy (eV)
PCB-28	256	186	16	258	186	24
$^{13}C_{12}$-PCB-28	258	198	23			
PCB-52	290	220	28	292	220	20
$^{13}C_{12}$-PCB-52	302	232	26			
PCB-101	324	254	26	326	256	18
$^{13}C_{12}$-PCB-101	336	266	24			
PCB-138	358	288	20	360	290	24
$^{13}C_{12}$-PCB-138	372	302	24			
PCB-153	362	290	20	360	290	24
$^{13}C_{12}$-PCB-153	372	302				
PCB-180	392	322	26	394	324	22
$^{13}C_{12}$-PCB-180	406	336	25			

S-1 GC-MSMS method validation

Analytical methods were fully validated in agreement with the guidelines laid down by SANTE/11312/2021 (Guidance document on analytical quality control and method validation procedures for pesticide residues and analysis in food and feed), following the conventional validation approach required for quantitative confirmation. The following parameters were evaluated: specificity, linearity, recovery, repeatability, and LOQ.

Specificity was assessed and verified in the blank matrix verifying the absence of signal higher than 30% of the LOQ level.

Linearity was studied by means of calibration curves within the range of 0,75 -100 ng/g for single congener (corresponding to 3–400 ng/g in the sample). Calibration curves were built by plotting the instrument signal versus the analyte concentration including zero level in the curve construction. Linear regression analysis was carried out and the linear calibration model was verified by correlation coefficients (Pearson's R) better than 0.992 and by Mandel test.

Recovery and repeatability were estimated by analyzing seven replicates at three concentration levels (6.26-125-250-400 μg/kg in the sample).

LOQ was estimated as the lowest spike level meeting the method criteria for recovery (80-120%) and repeatability (CV % ≤ 20%) (Table S2).

Table S2. GC-MSMS Method recovery, repeatability.

Compound	Nominal concentration (μg/kg)	Recovery%	Repeatability (CV %)
PCB-28	6.25<br>125<br>250<br>400	104.4<br>86.4<br>103.7	15.2<br>16.7<br>6.5
PCB-52	6.25<br>125<br>250<br>400	110.1<br>83.7<br>106.9	13.8<br>14.0<br>6.9
PCB-101	6.25<br>125<br>250<br>400	103.5<br>83.7<br>109.3	16.7<br>16.8<br>5.4
PCB-153	6.25<br>125<br>250<br>400	96.9<br>82.5<br>107	6.9<br>17<br>4.9
PCB-138	6.25<br>125<br>250<br>400	104.8<br>85.4<br>110.0	14.6<br>14.9<br>8.6
PCB-180	6.25<br>125<br>250<br>400	107.9<br>90.6<br>103.3	9.3<br>15.1<br>4.2
